# Enterovirus-like particles encapsidate RNA and exhibit decreased stability due to lack of maturation

**DOI:** 10.1371/journal.ppat.1012873

**Published:** 2025-02-04

**Authors:** Louis Kuijpers, Evdokia-Anastasia Giannopoulou, Yuzhen Feng, Wouter van den Braak, Abbas Freydoonian, Ramon Ramlal, Hugo Meiring, Belén Solano, Wouter H. Roos, Arjen J. Jakobi, Leo A. van der Pol, Nynke H. Dekker

**Affiliations:** 1 Department of Bionanoscience, Delft University of Technology, Delft, The Netherlands; 2 Intravacc B.V., Bilthoven, The Netherlands; 3 Moleculaire Biofysica, Zernike Instituut, Rijksuniversiteit Groningen, Groningen, The Netherlands; 4 Department of Physics and Kavli Institute of Nanoscience Discovery, University of Oxford, Oxford, United Kingdom; Purdue University, UNITED STATES OF AMERICA

## Abstract

To counteract hand, foot, and mouth disease-causing viruses such as enterovirus A71 and coxsackievirus A6, virus-like particles (VLPs) have emerged as a leading contender for the development of a multivalent vaccine. However, VLPs have shown rapid conversion from a highly immunogenic state to a less immunogenic state and low particle integrity lifetimes compared to inactivated virus vaccines, thus raising concerns about their overall stability. Here, we produce VLPs to investigate capsid stability using cryogenic electron microscopy (cryo-EM), mass spectrometry (MS), biochemical assays, and atomic force microscopy (AFM). In contrast to prior studies and prevailing hypotheses, we show that insect-cell produced enterovirus VLPs include encapsidated RNA fragments with viral protein coding sequences. Our integrated approach reveals that CVA6 VLPs do not undergo viral maturation, in contrast to virions; that they can encapsidate RNA fragments, similarly to virions; and that despite the latter, they are more brittle than virions. Interestingly, this indicates that CVA6 VLP stability is more affected by lack of viral maturation than the presence of RNA. Our study highlights how the development of VLPs as vaccine candidates should encompass probing for unwanted (viral) RNA content and establishing control of their maturation to enhance stability.

## Author summary

To counteract hand, foot, and mouth disease-causing viruses such as enterovirus A71 and coxsackievirus A6, virus-like particles (VLPs) have emerged as a leading contender for the development of a multivalent vaccine. Here, we produce VLPs to investigate capsid stability using cryogenic electron microscopy (cryo-EM), mass spectrometry (MS), biochemical assays, and atomic force microscopy (AFM). In contrast to prior studies and prevailing hypotheses, we show that insect-cell produced enterovirus VLPs include encapsidated RNA fragments with viral protein coding sequences. Our integrated approach reveals that CVA6 VLPs do not undergo viral maturation, in contrast to virions; that they can encapsidate viral RNA fragments, similarly to virions; and that despite the latter, they are more brittle than virions. Interestingly, this indicates that CVA6 VLP stability is more affected by lack of viral maturation than the presence of RNA. Our study highlights how the development of VLPs as vaccine candidates should encompass probing for unwanted (viral) RNA content and establishing control of their maturation to enhance stability.

## Introduction

Hand, foot, and mouth disease (HFMD) is becoming an increasingly concerning issue in the South-East Asian region [1]. Although vaccination campaigns against the conventionally most prevalent strain, enterovirus A71 (EV-A71), were initially successful, the emergence of other HFMD-causing viruses, most notably coxsackievirus A6 (CVA6), has tempered these successes. To combat the resurfacing of new disease-causing strains, the development of a multivalent vaccine would be highly beneficial. Virus-like particles (VLPs) have been proposed as a superior alternative to the conventional inactivated vaccines currently commercially available [2]. Due to their non-replicating and non-infectious nature, resulting from their proposed lack of genomic RNA, and their surface structure that is capable of triggering immune responses at similar or higher levels than their native virion counterparts, VLP vaccines offer substantial advantages [3].

CVA6 and EV-A71 are both viruses of the *Enterovirus* genus. All members of this genus have a positive sense RNA genome of ~7.5 kbp (kilobase pairs), consisting of a 5’ untranslated region (5’ UTR), a single open reading frame, and a polyadenylated 3’ UTR. The open reading frame encodes a 260 kDa polyprotein that comprises three regions P1, P2, and P3 [4,5]. P1 harbors the structural proteins that form the capsids, while P2 and P3 encompass the non-structural proteins responsible for the processing and replication of the viral RNA. Following translation and post-translational modification, the viral polyprotein undergoes a series of stepwise cleavage events, resulting in the generation of protein precursors, a subset of which exhibit distinct functionalities, such as proteolytic activity (e.g. the 3CD protein can cut the P1 region in the individual viral proteins VP0-VP1-VP3). Ultimately, this process concludes with the production of 11 mature viral proteins and the formation of stable pentamers, derived from the capsid proteins. This is succeeded by the assembly of procapsids (assembly intermediate), which, at the culmination of the replication process, can undergo conversion into the active virus (virion) [6].

Several studies have attempted to increase the thermostability of VLPs by subjecting virions to random mutagenesis during cultivation at elevated temperatures and then performing reverse engineering to introduce these mutations into VLPs [7,8]. This methodology has been successfully applied to poliovirus (PV) and enterovirus A71 (EV71) at laboratory scales, and could be adapted for other (enterovirus) VLPs [9–11]. The stability of the native configuration of enteroviruses relies on the presence of a lipid molecule, denoted the pocket factor, in a region at the base of the canyon within VP1 (hydrophobic pocket) [12]. Moreover, the conversion of their capsids from the highly immunogenic native state (D-antigen for PV; N-Ag for other enteroviruses) to the less immunogenic state (C-antigen for PV; H-Ag for other enteroviruses) is linked to dissociation of the pocket factor [13–15]. Although improved stability in these studies primarily refers to thermostability, which can prevent the conversion from N-Ag to H-Ag, thermostability does not necessarily imply other forms of stability. To date, no studies have investigated the differences in mechanical stability between the two candidate particles that are being used and developed for enterovirus vaccine purposes: the immunogenic N-Ag native virions and VLPs.

For enhanced safety, expression systems can replicate the natural morphogenesis of the virus to generate VLPs without the use of all viral proteins. An example of such a platform is the baculovirus expression vector system (BEVS), requiring only the expression the P1 polyprotein and 3CD protein to produce enterovirus VLPs [16–21]. The baculovirus expression vector system, which exploits *Baculoviridae* (baculovirus or BacV) to produce viral proteins of interest (or other proteins), exhibits a narrow host range. Although baculoviruses can enter mammalian cells, they are not pathogenic to humans, nor can they replicate in these types of cells [22]. This system offers not only relatively high yields and the potential for higher-order organism PTMs, but also an additional safety advantage.

Structurally, virions and VLPs differ in three major ways: First, native virions contain the full-length genomic RNA, whereas VLPs may either be empty or contain host cell proteins or mRNA–including the transcripts of the baculovirus expression vectors—and impact their stability [23]. While some VLPs mimicked after viruses other than the *Enterovirus* genus have been shown to carry random polynucleotides inside, enteroviruses seem atypical in this respect [24]. To date, the literature reports of a lack of RNA associated with enterovirus VLPs [25–28]. Second, native virions have the viral capsid protein VP0 cleaved into VP2 and VP4, whereas VLPs (generally) have VP0 intact. The mechanism responsible for VP0 cleavage remains currently unidentified, although it has been postulated that RNA may play a role in initiating this process. Third, native virions contain lipid pocket factors in the hydrophobic pockets of the canyons in VP1, whereas for VLPs produced in higher-order eukaryotes, initially contain a lipid pocket factor in a condensed configuration that can undergo transition into an expanded state with loss of the pocket factor [10,11,26]. The pocket factor stabilizes the capsid when in transit between cells and unbinding destabilizes it after attachment prior to uncoating [29,30]. VP0 cleavage and pocket factor rebinding represent the final stages of enterovirus morphogenesis *in vivo*, collectively termed viral maturation. Notably, pocket factor rebinding follows the cleavage of VP0 in enterovirus morphogenesis process (or vice versa), indicating a likely interconnection between these two processes [6]. All three differences significantly alter the mechanistic properties of the particles, potentially leading to variations in particle integrity lifetimes.

To investigate these possible differences, we performed a biophysical characterizations of the CVA6 VLP and their native virion counterpart. We used cryogenic electron microscopy (cryo-EM) to investigate the structural dissimilarities between VLPs and virions, biochemical assays to assess the ability of enterovirus-like particles to encapsidate RNA with viral protein coding sequences (vRNA), AFM nanoindentation experiments to explore the mechanical properties of VLPs and virions derived from two virus strains, and mass spectrometry to investigate the cleaving of the VP0. The integration of these four techniques enabled a comprehensive understanding of the biophysical properties and capabilities of enterovirus VLPs. Our results reveal that VLPs do contain RNA with viral protein coding sequences and that viral maturation is likely the cause of the increased stability of virions. This knowledge can serve as a foundation for the development of future stable vaccines.

## Results

**Fig 1:**
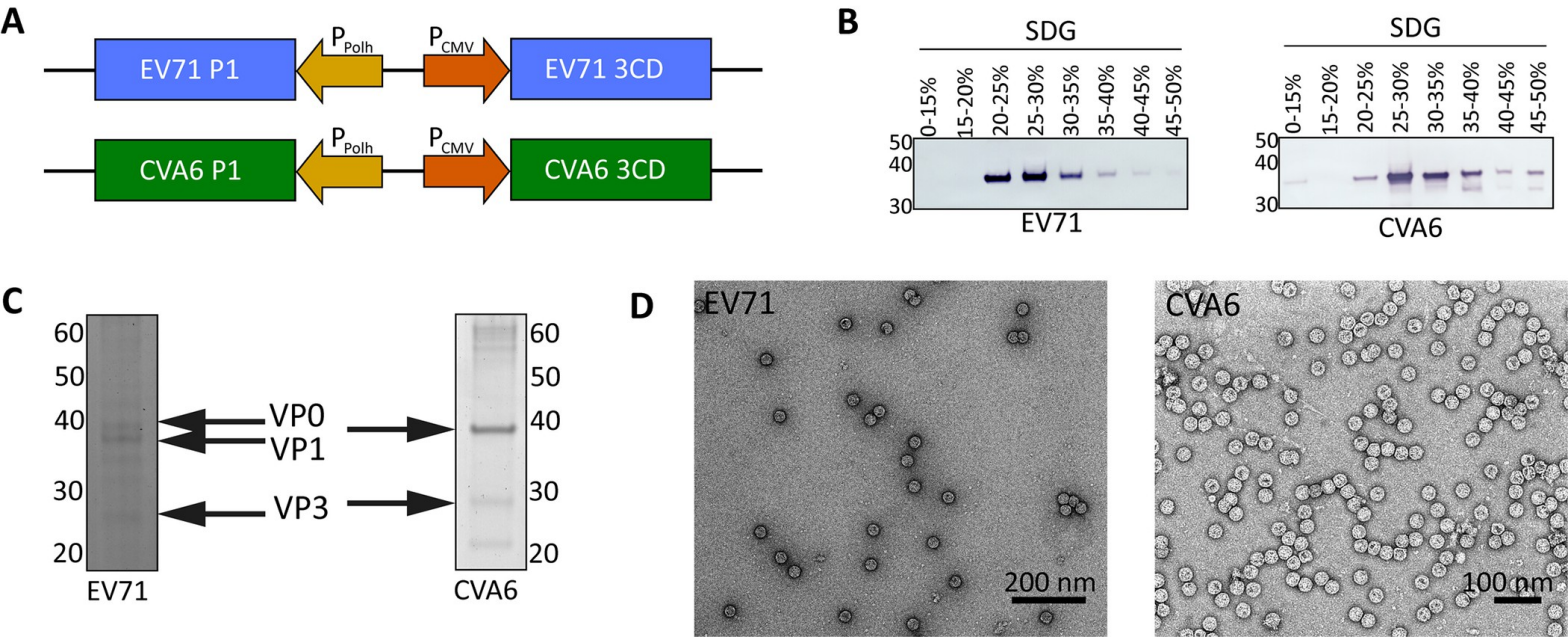
Production and evaluation of EV-A71 and CVA6 VLPs. (A) Construct design for EV-A71 and CVA6 VLP production using the baculovirus expression vector system and a previously described construct[31]. In short, both the P1 region, which encodes for the structural proteins, and the 3CD protease of the EV-A71 and CVA6 virus origins were inserted behind the polyhedrin and CMV promoters, respectively. (B) Western blot against the VP1 proteins of EV-A71 and CVA6. Samples originated from sucrose density gradient (SDG) fractions harvested and pooled based on sucrose percentage. For VLP production from both virus origins, the 25–30% SDG fractions were identified as most promising as they revealed the highest band intensities. (C) SDS PAGE results of the most promising SDG fractions for EV-A71 and CVA6 VLP samples. Both samples displayed protein bands for VP0, VP1, and VP3, albeit with dimmer bands for EV-A71. For CVA6, the bands for VP0 and VP1 overlap due to their highly similar molecular weights (36 kDa and 35 kDa, respectively), as can be observed by the increased intensity relative to other bands. (D) Negative stain electron microscopy images of EV-A71 (left) and CVA6 (right) VLPs.

In this work, we produced EV-A71 and CVA6 VLPs using a previously published baculovirus construct [31] (**Fig 1A**) together with optimized production and purification protocols to produce high yield batches of pure VLPs. Sucrose density gradient (SDG) ultracentrifugation was employed in the final step of purification to separate VLPs from contaminants. Based on anti-VP1 western blot analysis, the fraction with the highest concentration of viral proteins was determined to be 25–30% sucrose (**Fig 1B**). Subsequently, the purity of this fraction was evaluated by SDS PAGE analysis. This revealed few protein bands other than the expected ones encoded in our baculovirus vector, suggesting high purity of the VLPs (**Fig 1C**). Moreover, the produced viral proteins were individually identifiable, both for EV-A71 VLPs and CVA6 VLPs (**Fig 1C** left and right, respectively), despite the former being detected at significantly lower intensity. Evaluation of the CVA6 VLP gel revealed overlapping bands for VP0 and VP1, consistent with their highly similar molecular weights (36 kDa and 35 kDa, respectively)[26]. This difference is more pronounced for the EV-A71 viral proteins, allowing for the distinct observation of individual bands in this case. Negative stain electron microscopy analysis confirmed the high purity of both the EV-A71 and CVA6 VLPs, as backgrounds signals were negligible and protein aggregates were absent (**Fig 1D**).

**Fig 2:**
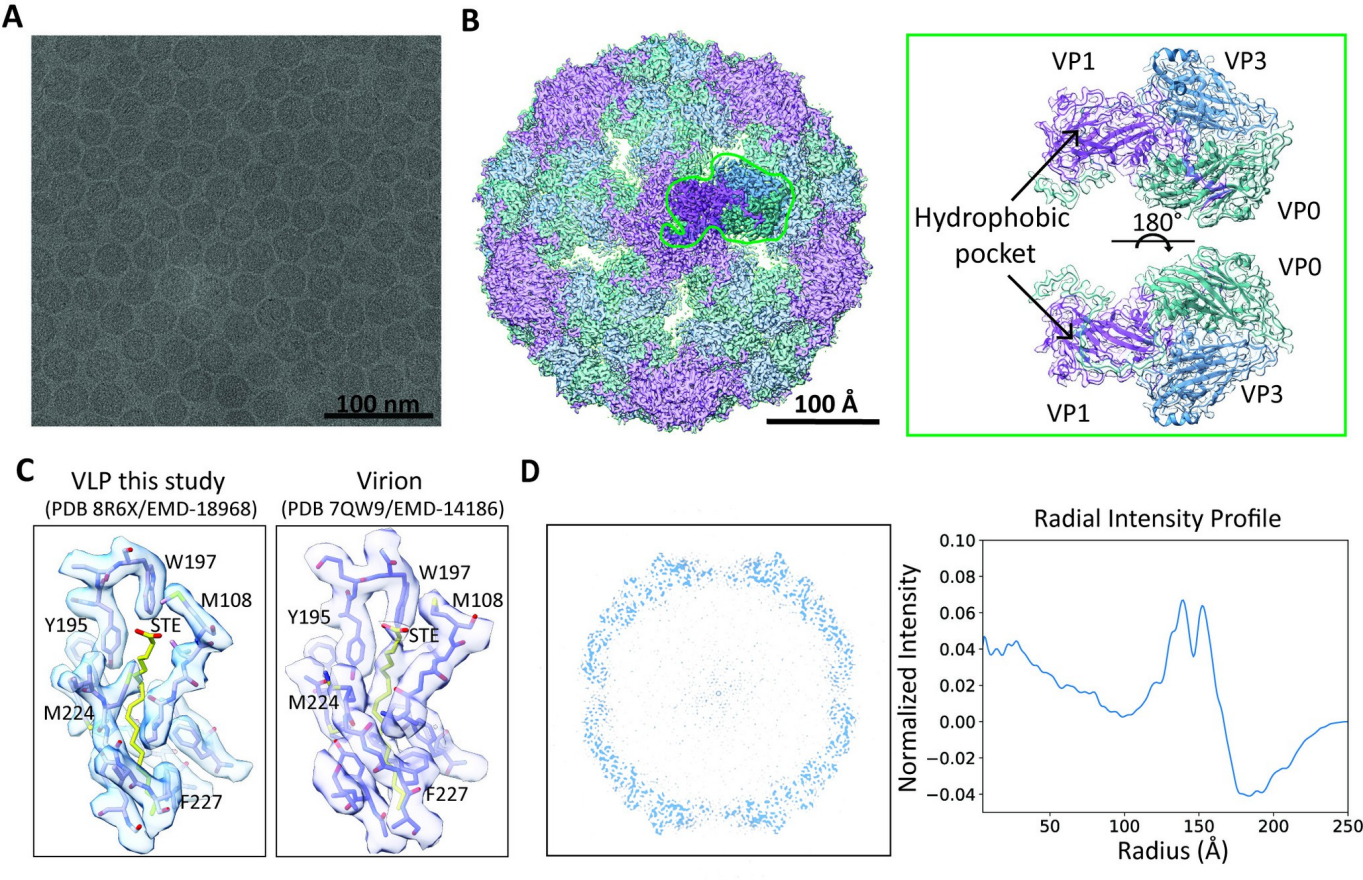
Cryogenic electron microscopy analysis of CVA6 VLPs: absence of pocket factor and increased electron density at the particle’s center. (A) Representative cryo-EM micrograph of CVA6 VLPs. (B) Cryo-EM structure of CVA6 VLPS viewed along the five-fold axis. The surface representation is segmented and colored by VP chain (VP0 = green; VP1 = purple; VP3 = blue). A single asymmetric unit (ASU) is highlighted. A close up of the ASU in two viewing orientations is also shown. (C) Comparison of the hydrophobic binding pocket on VP1 of our VLP structure (left) and the CVA6 virion[14] (right) showing the absence or presence of the pocket factor. (D) Radially averaged intensity profile of CVA6 VLP 3D density map, showing that there is higher intensity of something in the center.

We then proceeded to biophysical studies of these VLPs, starting with cryo-EM to examine their structure. Here, because the yield of CVA6 VLPs was consistently higher than that of EV-A71 VLPs, we focused on CVA6 VLP to obtain their high-resolution 3D reconstruction. This allowed us to firstly, compare our CVA6 VLP to previously reported structures of CVA6 VLPs, virions, and procapsids; and secondly, confirm the lack of viral maturation in VLPs as previously demonstrated [26,27]. Single-particle analysis with 44,582 particles yielded an icosahedral CVA6 VLP capsid structure at 3.15 Å overall resolution (**Fig 2A** and **Fig A in S1 Text**). The structure of the CVA6 VLP revealed typical surface features associated with enteroviruses, such as the prominent star-shaped plateau (mesa) at the 5-fold-symmetry axes, a narrow canyon-like depression surrounding the mesa, and an open channel situated at the 2-fold axes (**Fig 2B** and **Fig A in S1 Text**)[26]. Furthermore, the cryo-EM reconstruction revealed that the independent precursors of the particles (protomers) consisting of the three viral proteins (VP0,VP1, and VP3) were highly similar to the previously reported structure[26] (**Fig 2B** and **Fig B in S1 Text**). However, in our reconstruction we noted differences in the size of the particles and in the openings along the 2-fold axes (**Fig 2B** and **Fig B in S1 Text**). Further analysis using the root-mean-square deviation of atomic positions (RMSD) indicated that these differences were minor. The structure of our CVA6 VLP matched well with those of the CVA6 procapsid (RMSD between 189 pruned atom pairs: 0.729 Å; across all 207 pairs: 1.181 Å) [32] and the previously reported CVA6 VLP (RMSD between 196 pruned atom pairs: 0.917 Å; across all 221 pairs: 1.354 Å) [26] (**Fig B in S1 Text**). Despite these minor differences between the previously published CVA6 VLP structure and ours, both structures indicated an absence of the pocket factor (**Fig 2C**). This provides a first indication of a lack of viral maturation in these particles. The second indication hereof, also deduced from the cryo-EM structure (**Fig 2B**), was the absence of the characteristic VP0 cleavage into VP2 and VP4. This absence of cleavage concurred with both the structural features of the CVA6 procapsid and the previously reported CVA6 VLP structure [26,27].

We separately investigated the issue of viral maturation in the CVA6 VLPs using mass spectrometry (MS). With a sequence coverage of >80%, our MS data unequivocally revealed the presence of this concatenated VP0 protein and indicated extremely low concentrations of VP4 and VP2 (**Fig C in S1 Text**). Both VP4 and VP2 terminal peptides were ~3 orders of magnitude lower in response compared to the VP0 proteotypic peptide. Thus, on the basis of both our cryo-EM and MS findings, we conclude that viral maturation did not occur at a significant level in our VLPs.

Our 3D reconstruction and associated radial intensity averages reveal weak, but unstructured, density signal in the particle interior (**Fig 2D** and **Fig A and B in S1 Text**). Additional analysis of the center cross section of these particles confirmed this signal (**Fig 2D**). Such an increase in electron density within viral capsids could be associated with the presence of nucleic acids or large proteins [24,33]. Among individual classes, we observed spatial variation in such increases of electron density, suggesting that the underlying structure is dynamic in nature, shifting in position, orientation, or both. Cryo-EM data processing will tend to average out such dynamic behavior, precluding resolution of the underlying structure. While previous enterovirus VLP reconstructions did not detect the presence of RNA (center is masked out in the CVA6 structure) [26], we consider the presence of RNA to be the most plausible explanation for this observed increased density, whereby this RNA could change conformation or orientation within the VLP particle. This hypothesis is supported by the fact that RNA is readily available as a transcription product from the BacV vector and that some VLPs are able to attract random polynucleotides to the inside of the particles through electrochemical interactions [24].

**Fig 3:**
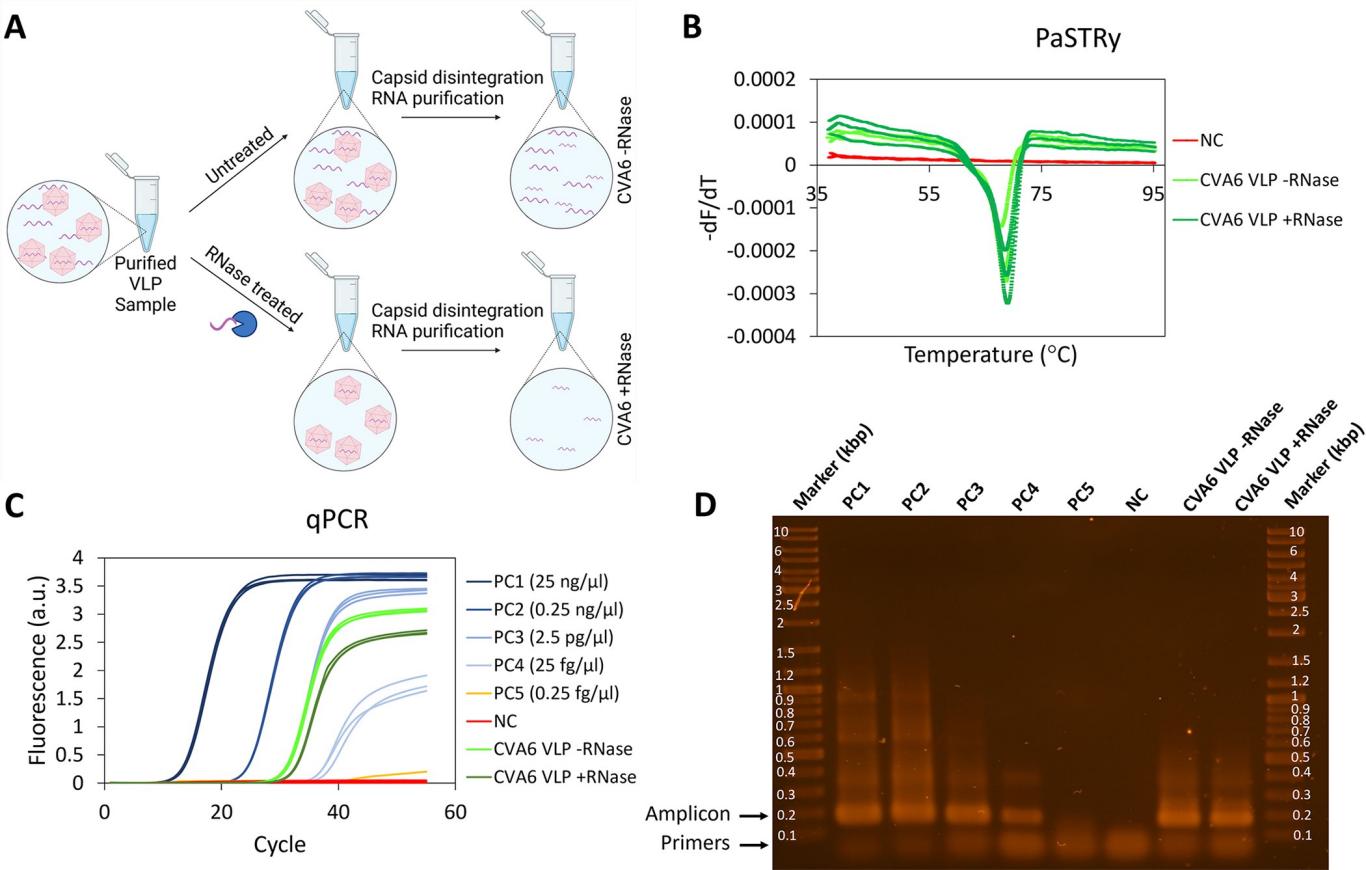
Detection of RNA presence in CVA6 VLPs. (A) Schematic representation of the PaSTRy and RT-qPCR assay used to probe for the presence of RNA outside the VLP capsids. The VLPs were divided into two samples: the first was treated with RNAse to eliminate any RNA outside of the particles, while the second remained untreated. For the PaSTRy assay, particle disruption is accomplished through temperature elevation, aiming to pinpoint the precise moment of disruption. For the qRT-PCR, both samples were chemically disrupted to extract any RNA present from the capsids, after which this RNA was purified. If VLP production were to result in a substantial presence of RNA exterior to the capsids, one would expect to observe a substantial difference in the RT-qPCR signal between the RNase-treated and untreated samples. Figure produced with Biorender. (B) PaSTRy results, depicted as the conventional melting curve (fluorescence change with respect to temperature), for both RNase-treated (dark green) and untreated (light green) CVA6 VLPs indicated a distinct peak starting at ~60°C, which correlates with the temperature for enterovirus genome release. This indicated that purified CVA6 VLP samples contained RNA. Negative controls that monitored the nucleic acid specific SYBR Green dye (red) indicated no signal, thereby confirming the absence of RNA presence. (C) RT-qPCR on untreated (light green) and RNase-treated (dark green) CVA6 VLPs with CVA6 specific primers indicated the presence of CVA6 RNA. Positive control (PC), comprising a plasmid containing the coding sequences for P1 and 3CD, was used in five serial 100-fold dilutions (dark-light blue, and orange). Negative control, devoid of viral RNA or DNA to validate the correct execution of the assay, is in red. (D) The products of the RT-qPCR reactions were loaded onto a 1.0% agarose gel. Positive controls PC dil.1 –PC dil. 4 indicated the 176 bp amplicon (indicated with arrow). The positive control with the highest dilution (PC dil.5) and the negative control did not indicate this amplicon. Both the RNase-treated (CVA6 +RNase) and untreated (CVA6 -RNase) CVA6 VLP samples also showed this amplicon at the same band intensity, indicating a highly similar concentration of RNA. All samples indicated the primer pairs at the bottom of the gel (indicated by arrow).

To investigate whether CVA6 VLPs contain RNA, we employed a particle stability thermal release assay (PaSTRy) [34]. First to ensure that the potentially detected RNA signals originated from inside the particles, we treated the main batch of CVA6 VLP samples with RNase (**Fig 3A**), while leaving a separate sample untreated as a control. This treatment ensured the elimination of any RNA present in the environment surrounding the CVA6 VLPs (**Fig 3A**). Next, the PaSTRy assay (**Fig 3B**) detects genome release through the binding of a fluorescent dye (SYBR Green) to nucleic acids released from the capsids upon temperature elevation. As the temperature was elevated to 60°C and higher, a major fluorescence peak was observed (**Fig 3B**), originating from RNA binding to the fluorescent dye and the accumulation of signal. Notably, the observed temperature threshold was consistent with the previously observed release temperature of viral genomes for enteroviruses [35]. In this way, we could establish that the VLPs harbored RNA. As SYBR Green binds RNA indiscriminately, the assay could not, however, discern whether the RNA present in the capsids was viral or host cell-derived in origin.

To independently verify the PaSTRy results and investigate the origin of the detected RNA, we next performed RT-qPCR experiments in which VP1-specific primers that target a part of the P1 region of the CVA6 genome are employed. Consequently, the presence of RNA with viral protein coding sequences (vRNA) in this assay should result in the presence of a 176 bp PCR-product (amplicon). Thus, both RNase-treated and untreated samples were subjected to chemical capsid disruption, any RNA present was isolated and purified (**Fig 3A**), and RT-qPCR carried out. Signals were observed for positive controls (**Fig 3C**, PC1-PC4, blue), the RNase-treated CVA6 VLPs (**Fig 3C**, dark green triplo curves), and untreated CVA6 VLPs (**Fig 3C**, light green triplo curves). This observation signified the presence of P1 vRNA in these samples. Only the positive control experiment with the most substantial dilution of plasmid containing the P1 region (**Fig 3C**, PC5, orange curve) did not yield observable signal, which we presume simply resulted from the signal falling below the detection limit. As expected, negative controls did not show detectable signal (**Fig 3C**, red curve). Another noteworthy observation was the delayed surpassing of the cycle threshold of the RNase-treated sample (**Fig 3C**, dark green triplo curves) in comparison to the untreated sample (**Fig 3C**, light green triplo curves). This implied the release of a low amount of RNA from the capsids, into the surrounding environment, likely as a consequence of VLP disintegration over time.

To exclude the possibility that some of the signal originated from primer pairs hybridizing with each other and amplification rather than detection and amplification of the desired region (amplicon), particularly e.g. in samples showing relatively low fluorescence signal (**Fig 3C**, light blue and both green curves), we additionally visualized the RT-qPCR product on a 1.0% agarose gel (**Fig 3D**). The gel revealed the presence of the 176 bp amplicon for both the CVA6 VLP samples (with or without RNase treatment) (**Fig 3D**, CVA6 -RNase and CVA6 +RNase). Furthermore, the same 176 bp amplicon was present in in all positive controls, expect for the final one, as previously described (**Fig 3D**, PC1 –PC5). Conversely, this amplicon was notably absent in the negative control (**Fig 3D**, NC). The additional band at the lowermost region of the gel, situated well below the 0.1 kbp marker (**Fig 3D**), revealed the presence of the primers. Taken together, the positive fluorescent signal in **Fig 3C**, as well as the presence of a 176 bp amplicon in **Fig 3D** for both the RNase-treated and untreated samples, corroborated the finding obtained through the PaSTRy, thus confirming the presence of RNA within the capsids. Additionally, the minor differences observed in the cycle threshold values (**Fig 3C**) and band intensities on gel (**Fig 3D**), which are indicative of the RNA quantity, between the two samples provide further evidence that the majority of the RNA originated from within the capsids. Crucially, these findings underscore the importance of investigating the presence of viral RNA (fragments) in VLPs, as the primers used are specific to the P1 gene, which is also present in the wild-type virus.

To further investigate the content of the CVA6 VLPs, mass photometry experiments were performed (**Fig F in S1 Text)**. The results revealed a broad distribution, indicating heterogeneity in the VLP content. Four populations were identified (red Gaussian fits) which approximately correlated with the molecular weights of the empty particle (~3.8 MDa) and particles containing RNA fragments coding for proteins 3C (~0.34 MDa), 3D (~0.86 MDa), and the P1 region (~1.59 MDa). Differences observed between the theoretical value (~5.4 MDa) and peak of the Gaussian fit (~5.1 MDa) for the P1 peak may have arisen from the Gaussian fits applied to the data and the limited statistical sample size. Alternatively, the differences between the four found molecular weights could have arisen from the various cleavage products of P1 or filling with the intact 3CD product (~1.2 MDa).

**Fig 4:**
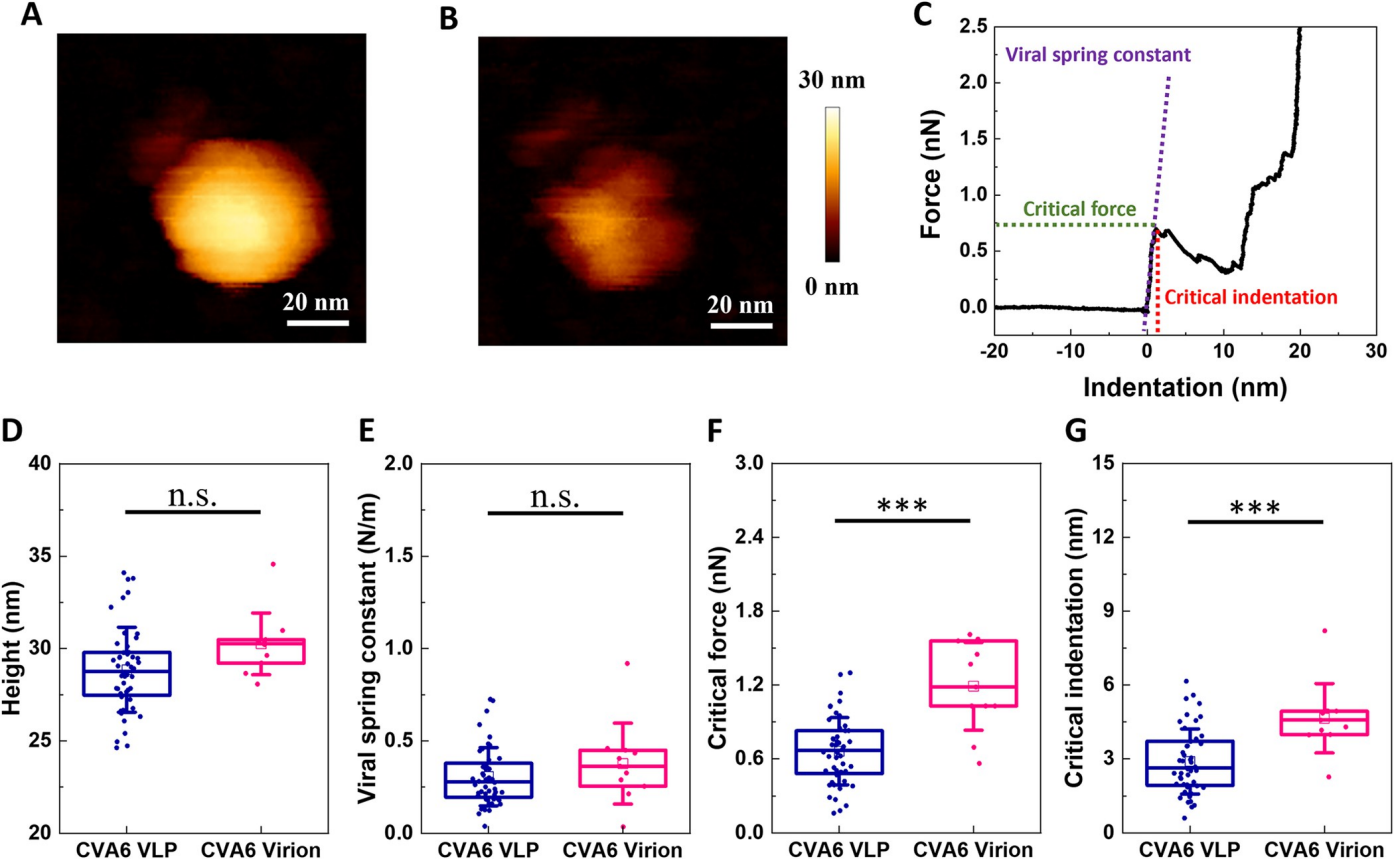
Comparison of mechanical properties of CVA6 VLPs and virions via AFM. (A) Example AFM image of an CVA6 VLP prior to nanoindentation. (B) The same CVA6 VLP after nanoindentation. (C) Force-indentation curve on CVA6 VLP obtained by AFM, highlighting the critical force (green in nN) and the corresponding critical indentation (red in nm). The spring constant was obtained as described previously [47] by first fitting the initial part of the force-displacement curve (purple) to a linear function to yield the total spring constant of the cantilever-particle system. The spring constant of the cantilever was separately obtained from calibration prior to each experiment [47]. (D) Heights of VLPs (blue) and virions (violet) as determined by AFM measurements. (E) Viral spring constant (N/m) for VLPs (blue) and virions (violet). (F) Critical force (nN) for VLPs (blue) and virions (violet). (G) Critical indentation (nm) for VLPs (blue) and virions (violet). P values are indicated by asterisks: p<0.001 (***), n.s.: not significant.

Having demonstrated that CVA6 VLPs contain RNA, we next wondered whether its presence could influence capsid stability, as has been postulated previously [36–39]. To probe this, we compared the mechanical properties of VLPs and virions using AFM nanoindentation, which has previously been used to study structures at both the micro and nanoscale [40] and reported on the mechanical properties of lipoproteins, bacteriophages, and protein capsids [41–44]. First we used the AFM imaging mode to visualize capsids before (**Fig 4A**) and after (**Fig 4B**) nanoindentation in order to determine their status (intact, buckled, broken, collapsed, etc.). Then we used the AFM force spectroscopy mode to directly apply a force to the capsids using the AFM cantilever (nanoindentation). This allowed us to probe the ability of capsids to withstand such a force as deduced by monitoring the corresponding extent of capsid deformation. At capsid failure (corresponding to a loss of structural integrity), we deduced the corresponding critical force and critical indentation (**Fig 4C**). These experiments were carried out for both CVA6 virions and VLPs (**Fig 4**). In addition, to identify whether the results were conserved across the *Enterovirus* genus, they were repeated on virions and VLPs originating from enterovirus A71 (**Fig D in S1 Text**).

It has been hypothesized that the viral maturation process, characterized by a reorganization of the viral capsid proteins, results in a condensation of the particle [45]. This condensation is thought to result in an up to 5% decrease in size of mature virions compared to expanded state VLPs [14,26,27,33,46]. As we did not observe signatures of viral maturation in our VLPs (**Figs 2C** and 3), their capsid sizes could be expected to be larger than those of the corresponding virions. Analysis of AFM images indicated that both VLPs and virions, regardless of their origin, had a similar average diameter of approximately 29 nm (**Table A in S1 Text**; **Fig 4D** and **Fig D in S1 Text**). However, given that the error bars are roughly 5% of the total size, possible differences in size fall within the experimental error.

From the subsequent nanoindentation experiments, we could determine several parameters that quantify the mechanical properties of VLPs and virions. It is imperative to note that our virions underwent inactivation through formaldehyde treatment. However, we have established that this process did not significantly alter their physical properties (**Fig E in S1 Text**). We first determined the viral capsid spring constant, an indicator of the stiffness of the particle (and defined as the slope of the linear response regime between externally applied force and particle deformation). The spring constant was calculated using previously established methodology [47] (see Methods). For CVA6, the average spring constants were determined to be 0.4 ± 0.2 N/m for virions (**Fig 4E** blue and **Table A in S1 Text**) and 0.3 ± 0.2 N/m for VLPs (**Fig 4E** violet and **Table A in S1 Text**). Statistical analysis indicated no significant difference between the virions and VLPs in regard to their mean spring constants.

For any material, the Young modulus (*E*) is deduced from the slope of the linear regime between stress and strain and is a size-independent parameter, in contrast to the spring constant. To convert the spring constant to the Young’s modulus, we utilized a published methodology [47,48] that assumes that the capsid is a spherical shell undergoing small deformations (i.e., in this idealized scenario, the particle is assumed to be made of a homogenous material with a constant radius and shell thickness [49]), resulting in the relationship $k_{cap}=\frac{\alpha Eh^{2}}{R}$, where *h* is the shell thickness, *R* is the outer radius, and *α* is a proportionality factor dependent on the geometry of the particle. To employ this formula, we assumed a value of 1 for *α* (shown to be a reasonable approximation for various capsids [42,47,48,50]). The outer radius was obtained by the AFM for each particle independently and shell thickness were obtained from the Virus Particle Explorer database [51]. This allowed us to deduce values of the Young’s modulus 0.2 ± 0.1 GPa (CVA6 virions) and 0.2 ± 0.1 GPA (CVA6 VLPs). These values are both similar to each other, as expected from the similarity of the corresponding spring constants, and to previously reported values for Hepatitis B virions and CCMV empty capsids [48,52].

Lastly, we examined the propensity to structural failure of the CVA6 VLPs and virions by determining the critical force that could be applied to them (**Fig 4C**, green) and the extent to which they could be indented (critical indentation; **Fig 4C**, red). Here, we found significant differences between virions and VLPs. Virions (**Fig 4F**, violet and **Table A in S1 Text**) exhibited a higher critical force than VLPs (**Fig 4F**, blue and **Table A in S1 Text**), indicating their ability to withstand higher forces before failure. Additionally, we observed a higher critical indentation for virions (**Fig 4G**, violet and **Table A in S1 Text**) compared to VLPs (**Fig 4G**, blue and **Table A in S1 Text**). This suggests that virions can undergo substantially greater deformation than VLPs before reaching a point of structural failure. From the AFM measured height, spring constant, critical force and critical indentation, consistency was observed across the *Enterovirus* genus, with EV-A71 exhibiting the same trends as CVA6 (**Fig D in S1 Text**).

## Discussion

Enterovirus virions and virus-like particles are thought to differ in three primary ways: the presence of RNA, the cleavage of VP0 into VP2 and VP4, and the occupation of the hydrophobic pocket by a pocket factor [14,26,27,33,46]. These three differences are thought to impact capsid stability and influence viral maturation[15,53–55]. For example, the presence of RNA is generally regarded to enhance the stability of the viral capsids, likely through capsid-RNA interactions, as observed in several *Picornaviridae* [14,56,57]. To obtain a comprehensive understanding of these three distinctive differences, including their influence on particle stability and thus potentially integral lifetimes, we performed structural, biophysical, and biochemical characterization of CVA6 and EV-A71 VLPs.

Surprisingly, PaSTRy and RT-qPCR results (**Fig 3A–3D**), together establish the presence of RNA fragments inside CVA6 VLPs, which is consistent with weak additional, albeit non-interpretable, density in the interior of the VLP cryo-EM density indicative of encapsulated/encapsidated material. It needs to be noted that the RNA, which may originate from multiple sources including host-cell and BacV transcripts, did not clearly bind the capsid or form a coat on its inside, as is observed in virions[14] (**Fig B in S1 Text**). However, we cannot exclude that only a fraction of the particles encapsulated RNA in a more stable manner, and this phenomenon might be averaged out in the cryo-EM analysis. Nonetheless, a more plausible scenario is that the P1 RNA either does not constitute the RNA-capsid interactions or is too short to sustain stable binding to the capsid. Nonetheless, the presence of vRNA fragments in the capsids raises uncertainty regarding the first hypothesized difference between enterovirus virions and VLPs. This finding contrasts with previous reports on other enterovirus VLPs such as poliovirus VLPs, and specifically with the previous structure determinations of CVA6 VLPs [25–28]. Because none of these studies indicated any density at the center of the particles and biochemical experiments investigating their content were not conducted, they did not produce evidence either for or against the presence of host cell mRNA or vRNA. Potential explanations for the observed differences in RNA presence and particle structure between the current study and previous works include variations in reaction conditions, differences in expression platforms or production organisms, and the absence of in-depth biochemical analysis. Our discovery of vRNA fragment presence within an enterovirus VLP is important because it demonstrates that, depending on the preparation conditions, enterovirus VLPs can indeed contain internal components. Although no viral RNA fragments are detected in our production process, the presence of such fragments in any other production platform can raise concerns about potential harboring and internalization of infectious RNA. Therefore, this necessitates rigorous evaluation of VLPs before they can be considered viable candidates for vaccine development.

Moving onto the two signatures of viral maturation, our analysis of our CVA6 VLPs using cryo-EM (**Fig 2B**) and MS (**Fig C in S1 Text**) revealed that both of these signatures were absent. In our CVA6 VLPs, VP0 remained intact, and the EM density for the pocket factor was absent. These structural features are fully consistent with previous work [26]. Consequently, the second and third postulated distinctions between virions and VLPs are confirmed.

It has been hypothesized that the presence of RNA can trigger viral maturation. It is known that in enteroviruses many of the residues that play a crucial role in viral maturation are located in the P1 region, which is encapsidated in a subpopulation of our VLPs (**Fig F in S1 Text**). For example, in poliovirus, conserved residues 195H (VP2) and 194P (VP2), located near the scissile bond, play a vital role in promoting VP0 cleavage [13,58,59]. Similarly, residue 107H (VP1) is proposed to regulate VP0 maturation in EV-A71 [60]. The present work shows that RNA coding for the CVA6 P1 region is available and packaged into the CVA6 VLPs (**Fig 3C,D**) [61]. Taken together with the lack of observable VP0 cleavage and binding of a pocket factor in VLPs (**Fig 2B and Fig C in S1 Text**), this leads to the conclusion that the presence of P1 vRNA alone is insufficient to complete the viral maturation process. This suggests that CVA6 vRNA may not be the sole factor responsible for triggering viral maturation and that other processes must be involved. This contrasts with findings obtained for other enteroviruses: for example, for hand, foot, and mouth disease virus it has been demonstrated that viral maturation is dependent on genomic RNA and occurs only when the capsid proteins are in contact with it [36,37,62]. These different findings indicate that further research is required to comprehend the role of vRNA and other factors in the maturation of virus capsids more fully.

Lastly, we looked into the impact of RNA presence or viral maturation on the mechanical properties of viral capsids using AFM nanoindentation experiments. Our results showed that the CVA6 VLPs and CVA6 virions have identical Young’s moduli in both their mean and standard deviation, namely 0.2 ± 0.1 GPa (**Table B in S1 Text**). This value is comparable to those reported for other viruses, such as cowpea chlorotic mottle virus (CCMV) [48], hepatitis B virus (HBV)[52], immature murine leukemia virus (MLV)[63], and Norwalk VLPs [64]. In contrast, it is significantly lower than the Young’s modulus measured for bacteriophages λ and Ф29 [42,65]. Substantial differences in mechanical characteristics between dsDNA bacteriophages and viruses that self-assemble around their genome have been previously reported and, are suggested to be attributable to differences in capsid assembly pathways [52]. Bacteriophages have an active, ATP-driven packaging machinery for their DNA, whereas CCMV and MLV self-assemble while simultaneously integrating their RNA into the capsid. In the self-assembly scenario, the RNA is unlikely to exert high pressures onto the viral capsids, as it is not forcefully introduced into the particles. The present results on CVA6 fit into this picture.

The observed differences in critical force (**Fig 4F**) and critical indentation (**Fig 4G**) indicate that CVA6 VLPs can withstand less force and deformation prior to structural failure than their virion counterparts. These findings might *a priori* be interpreted as implying that the presence of RNA in virions provides additional stabilization to their capsids. However, as we have shown that CVA6 VLPs also contain vRNA fragments (**Fig 3C and 3D**), the mere presence of vRNA is not sufficient for stabilization. A likely alternative explanation is that the viral maturation (**Fig 2A and 2B**, and **Fig C in S1 Text**) induces the enhanced stability of virions over VLPs (**Fig 4F–4G**). Viral maturation as a trigger for capsid stabilization is in agreement with the understanding that the attachment of the pocket factor to virions enhances capsid stability during intercellular transit and that its detachment results in destabilization after cell attachment but prior to uncoating[29,30,66].

Overall, the reduced stability of VLPs has significant implications for their use as vaccines. To achieve comparable stability to that of virions, artificial methods of inducing viral maturation or alternative means of stabilizing the capsids must be explored. Ultimately, this may be a critical factor in the success of VLP vaccines in the future.

## Materials & methods

### Construction of recombinant baculoviruses

Previously reported construct optimizations for the production of enterovirus A71 (EV-A71) indicated that the highest viral protein yields were obtained using the P1 and 3CD coding sequences behind the polh and CMV promoters, respectively [31]. Two transfer plasmids, containing the aforementioned genes originating from EV-A71_C4 and CVA6 [67], respectively, were amplified using *E*. *coli* JM109 competent cells (Promega) and isolated using NucleoBond Xtra Midi EF (Macherey-Nagel). The amplified transfer plasmids were verified by double digestion using multiple restriction enzyme pairs. To construct the recombinant baculovirus, the transfer plasmid and *flash*BAC GOLD DNA (Oxford Expression Technologies) were co-transfected into Sf9 cells (Gibco). The two different baculovirus stocks for the EV-A71 and CVA6 origin viral proteins were designated BacV-EV-A71 and BacV-CVA6, respectively. The baculovirus was propagated over the course of three passages and titered by the end-point-dilution method using Sf9 Easy Titer (Sf9ET) (Kerafast) cells according to the manufacturer’s protocol.

### VLP production & purification

Production of EV-A71 and CVA6 VLPs was accomplished by infection of High Five cells (Gibco). Erlenmeyer culture flasks (1 l) with 400 ml working volumes of 1.0 * 10^6^ cells/ml Hi5 cells were infected by either BacV-EV-A71 or BacV-CVA6 (MOI 0.001 and 5, respectively) in Sf-900IISFM (Gibco). Subsequently, the following steps were carried out on each culture. First, the culture was centrifuged (Heraeus) at 3,000 rpm for 15 min at 4°C. The pellet was discarded, and the supernatant was supplemented with Triton X-100 (final concentration 0.1%) to prevent protein aggregation. The supernatant was double filtered using 0.45 μm and 0.22 μm filter (Millex). The filtered supernatant was subjected to sucrose cushion ultracentrifugation (30% W/W sucrose in PBS) for 5 h at 141,000 × g. The pellet was resuspended in 1 ml phosphate-buffered saline (PBS, Gibco) and subjected to another centrifugation (10,000 × g, 10 min), after which the pellet was discarded and the supernatant was applied onto a discontinuous sucrose density gradient (SDG) (15–45% W/W sucrose in PBS) and ultracentrifuged for 3 h at 141,000 × g (ThermoFisher Scientific). The SDG was harvested by 1 ml sampling from the top. Samples were pooled into 8 concentration ranges based on the percentage of sucrose (0%-15%, 15%-20%, 20%-25%, 25%-30%, 30%-35%, 35%-40%, 40%-45%, and 45%-50%), whereby the sucrose density was measured by refractometer (Hanna instruments).

### Virion production and purification

EV-A71 and CVA6 inactivated virions were provided by Intravacc B.V.

### SDS PAGE & Western blot

To confirm the viral protein production of EV-A71 and CVA6, VP1 (both origins) western blot analysis and SDS PAGE were performed. For this, 40 μl of each culture was mixed with 10 μl loading buffer (Intravacc) and heated at 100°C for 15 min. Then, 15 μl of each mixture was loaded on NuPAGE 10% Bis-Tris gel (Invitrogen) and electrophoresis was performed at 200 V for 45 min. The gel was washed three times for 5 min using purified water. Depending on the purpose of the gel, it was subjected to either staining or overnight blocking (see sections below).

### SDS PAGE

The gel was stained with Imperial protein stain (Thermo Fisher Scientific) for 1 h and destained in multiple cycles and overnight using purified water.

### Western blot

The proteins were blotted onto a nitrocellulose membrane (ThermoFisher Scientific) using a semi-dry blotting machine (Hoefer) run at 60 mA for 60 min. The membrane was washed two times in wash buffer (PBS + 0.1% W/W Tween 20 (Sigma-Aldrich)) and blocked overnight at 4°C (PBS + 0.1% Tween 20 (Sigma-Aldrich), and 0.5% Protifar (Nutricia)). Next, the membrane was incubated with 1:1,000 of either mouse anti-EV-A71-VP1 (Abnova, MAB1255-M08) or rabbit anti-CVA6-VP1 (ThermoFisher Scientific, PA5-112001) in block buffer for 90 min on a roller incubator. The membrane was washed five times using wash buffer and incubated with 1:2,000 of either goat anti-mouse IgG Human ads-AP (Southern Biotech, 1030–04) or goat anti-rabbit IgG Human ads-AP (Southern Biotech, 4050–04) in block buffer for 90 min on a roller incubator. The membrane was washed three times using wash buffer and once using purified water. The membrane was colored using AP conjugate substrate kit (BIO-RAD, 1706432). The colorization reaction was terminated by the addition of an excess of purified water.

### Atomic force microscopy

#### AFM sample preparation

Prior to AFM measurements, the stock solutions of the viral particles were diluted 8 times using phosphate buffered saline (PBS, Sigma-Aldrich). A hydrophobic glass cover slip was applied as the substrate to immobilize the viral particles during AFM measurements. The hydrophobic coating on the glass cover slip was obtained by applying a hexamethyldisilazane (Sigma-Aldrich) treatment as described previously [42,47]. A droplet of 20 μl diluted stock solution was deposited on this hydrophobic glass cover slip. After 15 min incubation at room temperature, another 800 μl PBS was added to a liquid receptacle for subsequent AFM imaging and nanoindentation.

### AFM imaging and nanoindentation

Imaging and nanoindentation experiments of the viral particles were conducted by AFM (NanoWizard, JPK). All experiments were performed in PBS. Before AFM imaging, the cantilever (qp-Bio AC CB2, Nanosensors) with a nominal spring constant of 0.1 N/m was calibrated using the contact-based method. The parameters for imaging both the virions and the VLPs in quantitative imaging (QI) mode were kept constant, and the setpoint for imaging was set at 0.07 nN.

After imaging, nanoindentation was performed at the center of a viral particle. The nanoindentation procedure was carried out according to a previously published protocol [47]. The settings for nanoindentation were kept constant and set as follows: Z length, 200 nm; setpoint, 3 nN; loading rate, 50 nm/s. Before and after indenting the targeted viral particle, indentations were performed on the glass substrate adjacent to the viral particle to confirm the cleanliness of the tip. Finally, the indented viral particle was imaged again to visualize the structural state of the particle following nanoindentation.

### Data processing

The viral spring constant of the particles was calculated using a previously published method [47], in which the initial part of the Force-Displacement (F-Z) curve obtained from AFM nanoindentation was fitted to a linear function. Its slope yielded the total spring constant of the cantilever-particle system, and the spring constant of the cantilever was obtained from the above described calibration [47]. As this fit yielded the total spring constant of the cantilever-particle system, the spring constant of the particle could then be extracted in conjunction with independent calibration of the cantilever [47].

The F-Z curve can be converted to Force-Indentation (F-D) curve using previously published methodology [47] in order to extract the critical force and critical indentation of the viral particle. The critical force was measured from the ordinate of a discontinuous point on an F-D curve, and the critical indentation was measured from the abscissa of the same discontinuous point.

Due to the convolution of the tip with the sample, the corrected height was used as the size of the measured viral particle. The corrected height was calculated using previously published methodology [47] that corrects for the imaging force-induced particle deformation. Unless specified otherwise, the heights mentioned are corrected heights.

To evaluate the similarity or difference of independently measured quantities in different experiments, the one-way ANOVA method of statistical analysis was conducted using Origin software. Unless otherwise noted, stated errors are standard deviation (SD).

### Electron microscopy

#### Negative stain

Negative stain transmission electron microscopy was employed for the direct visualization of VLPs. Of each DSP fraction, 3 μl sample solution was dispensed on glow discharged EM grids (Quantifoil, carbon-supported, Cu-400) incubated for 60 s and blotted away using filter paper. To increase the particle concentration on the carbon support this procedure was repeated three times. Next, grids were subjected to two wash cycles using 10 μl PBS. Excess liquid was blotted away using filter papers. Staining was performed using 2% uranyl acetate for 1 min. Excess liquid was blotted away using filter paper. Imaging was performed using a JEOL JEM-1400plus TEM operated at 120 kV and micrographs were acquired on a TVIPS F416 CMOS camera at multiple magnifications.

### Cryo-EM

#### Grid preparation

Quantifoil Cu-200 R 2/1 grids with a continuous carbon layer (2 nm) were glow discharged (Quorum GloQube,18 mA, 90 seconds). Immediately after glow discharging, 3 μl of CVA6 VLPs was applied to the grids, incubated for 3 min on the surface of the grid in a Leica GP2 vitrification robot at 99% humidity and 20°C before blotting for 10 s from the carbon side of the grid and then immediate flash-cooling in liquid ethane.

#### VLP data collection

VLP-containing grids were imaged on a JEM 3200FSC (JEOL), operated at 300 kV and with a 20 eV slit (centered at 0 eV) inserted below the omega energy filter. Images were recorded on a K2 Summit direct electron detector (Gatan) at a magnification of 40k, corresponding to a pixel size of 0.975 Å at the specimen level. Image acquisition was performed using SerialEM [68] and micrographs were collected with a defocus range of 0.5–2.5 μm and a cumulative electron exposure of 57.23 e/ Å^2^. A total of 643 micrographs were collected from a single grid, with a single shot performed per grid-hole.

#### VLP data processing

Image processing was performed with Cryosparc V4.2.1 [69]. Initially, patch motion correction was done to correct for stage drift and beam-induced movement, and then the selected micrographs underwent patch contrast transfer function (CTF) estimation and were manually curated to remove poor quality micrographs. Using a manual blob picker with a box size of 512 pixels, 200 particles were selected and subjected to 2D class averaging to generate references for the template picker tool. A total of 44,582 usable particles were finally selected after multiple iterations of 2D classification and particle picking. An initial model was generated *ab initio* from the selected particle set imposing icosahedral symmetry. This model was further refined using homogeneous refinement to result in an initial cryo-EM map at 3.6 Å resolution. The model was further improved by refining per-particle defocus, per-group CTF parameters and by using Ewald sphere correction, resulting in a final 3D volume at 3.15 Å overall resolution.

### RNA presence determination

#### Particle stability thermal release assay

Purified CVA6 VLPs were mixed 1:4 with SYBR Green II RNA gel stain (500X) in a final volume of 50 μl in a 96 well plate (Lightcycler 480 Multiwell, Roche). PCR was run with 0.03°C/s increments, 17 measurements/°C, with a temperature ramp from 37°C to 95°C. Measurements were taken using FAM green channel (520 nm). The negative control (NC) in this experiment consisted of a template-free sample. Data was analyzed using Microsoft Excel.

#### Reverse transcription and polymerase chain reaction

The purified CVA6 VLPs were split into two 150 μl samples. One of the samples was treated with 1:100 RNaseI (Thermo Fisher Scientific) to a final concentration of 0.1 U/ml, and incubated for 30 min at 37°C. Subsequently, both treated and untreated VLP samples underwent viral RNA isolation using the NucleoSpin RNA virus kit (Macherey-Nagel), as per the manufacturer’s protocol. The isolated RNA was subject to RT-qPCR using the LightCycler Multiplex RNA Virus master kit (Roche), using a custom running protocol (**Table 1**) and primers (**Table 2**). Of the PCR product, 15 μl was mixed with 3 μl 6X loading dye (NEB, Cat#B7024), and the entire 18 μl was loaded onto a 1.0% TAE agarose gel. The gel was run for 1 h at 100 V in TAE buffer, stained for 30 min in ethidium bromide (final concentration 0.25 μg/ml), and destained for 10 min in purified water. The positive controls comprised a serially diluted plasmid containing the P1 region and were employed not only to serve as an experimental procedure control but also to establish the lower detection limit. The negative control (NC) in this experiment consisted of a template-free sample. Data was analyzed using Microsoft Excel.

**Table 1:** RT-PCR running protocol.

Step	Temperature (°C)	Time (mm:sec)	Cycle	Ramp (°C/sec)
RT step	50	10:00	1	4.4
Denaturation	95	00:30	1	4.4
Denaturation	95	0:05	45	4.4
Annealing	56	0.10	45	-
Extension	60	0:30	45	2.2
Cooling	37	0:10	1	2.2

**Table 2:** Primers used for RT-PCR for the detection of viral CVA6 RNA.

Primer	Sequence (5’ ➔ 3’)
CVA6 Probe	[FAM]CGGCGCTGCTGCACGAATCCC[BHQ1]
CVA6 Fw	TACTCTAGGGCTGGTCTGGT
CVA6 Rv	TCGTTCAGGTTGGAGACGAA

### Mass spectrometry

Protein and peptide identification was performed in triplicate, using the bottom-up approach. Briefly, 10 μl of CVA6 VLP sample was incubated in 0.1% Rapigest SF (Waters Corporation, Milford MA, USA) and 100 mM phosphate buffer pH = 7.4 (Merck KGaA, Darmstadt, Germany) for 30 min at 80°C to denaturate the VLPs. Protein reduction and alkylation was accomplished with TCEP and Iodoacetamide (Thermo Fisher Scientific, Waltham MA, USA) essentially as described by the supplier’s protocol. Subsequently, proteolytic digestion was performed with 400 ng of sequencing grade trypsin (Promega, Madison WA, USA) and overnight incubation at 37°C. Rapigest was removed by the addition of TFA (Merck KGaA, Darmstadt, Germany) to a final concentration of 1 vol% and incubation at Room Temperature for 1 hr. After centrifugation, an aliquot of the clear supernatant was subjected to nanoscale reversed-phase liquid chromatography electrospray mass spectrometry [70]. Peptides were loaded on a trapping column (Reprosil-Pur C18-AQ 5 μm (Dr. Maish, Ammerbuch-Entringen, Germany); 20 mm long × 100 μm inner diameter, packed in-house) using solvent A (0.1 vol% Formic acid (Merck KGaA, Darmstadt, Germany) in water for 10 min at a column flow rate of 3 μl/min. Subsequently, the peptides were separated by reversed-phase chromatography on an analytical column (Reprosil-Pur C18-AQ 3 μm (Dr. Maish, Ammerbuch-Entringen, Germany); 35 cm long × 50 μm inner diameter, packed in-house) at a column flow rate of 125 nL/min. The gradient was started with 5 vol% solvent B (0.1 vol% Formic acid in acetonitrile (Biosolve BV, Valkenswaard, The Netherlands) to 45 vol% in 10 min, followed by a second increase of solvent B to 85 vol% in 5 min.

Mass spectrometric data was acquired on a Tribrid Orbitrap Fusion Lumos (Thermo Fisher Scientific, Waltham, MA; USA) equipped with a Nanospray Flex Ion Source. Full scan MS1 spectra were acquired with a scan mass range of 350–1,500 Th at 120,000 resolution (FWHM) with an Orbitrap readout and internal mass calibration performed on each scan. Precursor ions with charge states between 1–4 and intensities >10,000 counts were selected for Collision-Induced Dissociation (CID) fragmentation (MS2 scans) with a normalized collision energy of 35% and an Ion Trap readout. Targeted *m/z*-values for the tryptic peptides discriminative for VP0-cleavage, *i*.*e*. C-terminus of VP4, N-terminus of VP2 and the overlapping VP4-VP2 peptide (the latter indicative for intact VP0) were included to prioritize the MS2 scan on these proteotypic peptides.

Peptide and protein identifications have been performed with Peaks X (Bioinformatics Solutions Inc., Waterloo ON, Canada), using a concatenated protein database of VP0, VP1, VP2, VP3 and VP4 from CVA6. Dynamic modifications were deamidation of Asparagine (N) and Glutamine (Q) and oxidation of Methionine (M). A fixed modification was acetamidation of Cysteine (C). Semi-specific cleavage of Trypsin has been selected. Precursor and product ion mass tolerances were set at 3 ppm and 0.4 Da, respectively.

### Mass Photometry experiments

All mass photometry experiments were carried out at room temperature using a OneMP instrument (Refeyn). Sample wells were analyzed by adhering a 6-well silicone gasket to a clean cover glass. For each measurement, 18 μL of PBS was added to a new sample well, and the interface between the buffer and the cover glass surface was focused. Subsequently, 2 μL of sample was introduced into the same well. The binding of CVA6 VLPs to the cover glass surface was imaged and recorded for 2 minutes at a frame rate of 1 kHz. Each sample well was used only once. Reproducibility of the mass photometry measurements was validated through independent experiments using the same sample. All mass photometry images were processed and analyzed using DiscoverMP (Refeyn). The interference intensities of the CVA6 VLPs were converted to masses through calibration with known mass standards (MassFerence P2; Refeyn).

## Supporting information

The supplementary information consists of 6 supplementary figures and 3 supplementary tables describing:

The EM workflow. **Related to Fig 2.**

The structural comparison of our VLPs to published structures. **Related to Fig 2.**

The MS analysis. **Related to Fig 2.**

The mechanical parameters (spring constant, critical force, critical indentation, and Young modulus) measured by AFM. **Related to Fig 4**.

The AFM comparative measurements for EV71 to the CVA6 VLPs and virions. **Related to Fig 4**.

The AFM investigation into the influence of the inactivation process on the stability of the capsids. **Related to Fig 4**.

The numerical values from the AFM measurements and used for Young moduli calculations. **Related to Fig 4**.

The mass photometry data of our VLPs.

S1 Text **Fig A. Cryo-EM processing workflow in Cryosparc.** (A) Raw micrograph processing on CryoSPARC and steps required to proceed with 2D classification. (B) 2D classes of selected particles. (C) *Ab initio* reconstruction of cryo-EM density using Icosahedral symmetry. (D) Homogeneous refinement of map that resulted in 3.15 Å resolution. The final density map obtained can be viewed in cyan through its 5-fold and 2-fold symmetry axes. (E) Quality assessment metrics. (upper left) GSFSC resolution as computed from the processing software CryoSPARC, indicates a final map resolution of 3.15 Å resolution. (upper right) Local resolution of the final map plotted on the surface of the map as a colour gradient that ranges from 2.8–3.8 Å. (lower left) Euler angle distribution plot that shows the angular distribution of the particles (lower right) Model vs FSC plot evaluates the map-to-model fit match at 0.5 threshold. **Related to Fig 2. Fig B. Evaluation of the increased density at the center of the VLPs.** (A) Comparison of the cryo-EM density of the current study VLP (blue), EMD_6829 (yellow), EMD_6752 (green) and EMD_14186 (purple), shown from the face of the 5-fold axis, (1st column), the 2-fold axis (2^nd^ column), and the central slice of each particle (3^rd^ column). (B) Graphical plot of the normalized radial intensity of each particle density plotted against the radius (Å). (C) Comparison of the pdb models of the ASUs of the VLP originating from this study (blue), 5yhq (yellow), and 5xs5 (green) rotated along the x axis by 90° from left to right. **Related to Fig 2. Fig C. LCMS analysis assessing the cleavage of the viral protein VP0 as indicator of viral maturation.** (A) Full VP0 amino acid sequence, with VP4 and VP2 indicated in red and green, respectively. Bold and underlined indicates the concatenated VP4/VP2 sequence of the proteotypic peptide for VP0 upon digestion. For VLPs that underwent viral maturation, the C-terminal proteotypic peptide of VP4 (EVAAPLQ) and the N-terminal proteotypic peptide of VP2 (SPSVEACGYSDR) should be identified, while for immature VLPs, the concatenated sequence of these VP4-VP2 termini (EVAAPLQSPSVEACGYSDR) should be identified. (B) MS analysis revealed the presence of VP0 (concatenated VP4-VP2) in high abundance. Additionally, the C-terminal and N-terminal proteotypic peptides of VP4 and VP2, respectively, were identified, however in extremely low concentrations (~3 orders of magnitude lower than that of VP0). **Related to Fig 2. Fig D. AFM nanoindentation results for EV71, CVA6 virions and VLPs.** (A) Corrected height of the particles (EV71 VLPs (black), EV71 virions (red), CVA6 VLPs (blue), and CVA6 virions (violet)) as determined by AFM measurements. (B) Viral spring constant (N/m). (C) Critical force (nN). (D) Critical indentation (nm). P values are indicated by asterisks: p<0.001 (***), p<0.01 (**), and p<0.05 (*). N.s.: not significant. The CVA6 data is presented again for direct comparison with the EV71 data. **Related to Fig 4. Fig E. AFM particle stability parameters not affected by formaldehyde inactivation.** To investigate whether stabilizing effects of formaldehyde treatment accounted for the observed differences in the mechanical properties between virions and VLPs, a supplementary batch of CVA6 VLPs underwent the same formaldehyde treatment as the CVA6 virions, after which we again performed nanoindentation force spectroscopy experiments using the AFM. (A) Viral spring constant (N/m). CVA6 VLP (blue), CVA6 inactivated virions (violet), and CVA6 inactivated VLPs (green). (B) Critical force (nN). (C) Critical indentation (nm). P values are indicated by asterisks: p<0.001 (***), p<0.01 (**), and p<0.05 (*). N.s.: not significant. From these experiments, we concluded from this data that there was no significant difference between treated and untreated CVA6 VLPs. The same is assumed to hold true for EV71 VLPs. Therefore, it was concluded that the inactivation process did not have any additional stabilizing effects on the CVA6 virion capsids. **Related to Fig 4. Figure F. Mass photometry data on CVA6 VLPs.** To further investigate the content of the CVA6 VLPs, mass photometry experiments were performed. The results revealed a widespread distribution, indicating heterogeneity in the VLP content. Four populations were identified (red Gaussian fits) which approximately correlated with the molecular weights of the empty particle (~3.8 MDa) and particles containing vRNA fragments coding for proteins 3C (~0.34 MDa; Gaussian peak at 4.2 MDa), 3D (~0.86 MDa; Gaussian peak at 4.6 MDa), and the P1 region (~1.59 MDa; Gaussian peak at 5.1 MDa). Differences observed for the P1 peak may have arisen from the Gaussian fits applied to the data and the limited statistical sample size. Table A. Measured size and determined mechanical parameters for the different capsid structures. Related to Fig 4. Table B. Deduction of Young’s moduli from measured size parameters for the different capsid structures. Related to Fig 4. Table C. Processing and structural information of our CVA6 VLP.
(DOCX)

S1 Raw Data underlying Fig 3. (XLSX)
