# Supplementary material for: Enterovirus-like particles encapsidate RNA and exhibit decreased stability due to lack of maturation
Source: PLoS Pathog. 2025 Feb 4;21(2):e1012873. doi: 10.1371/journal.ppat.1012873 (PMC11793780; doi:10.1371/journal.ppat.1012873)

# Supplemental Information

**Enterovirus-like particles encapsidate RNA and exhibit decreased stability due to lack of maturation.**

**Louis Kuijpers^1,2^, Evdokia-Anastasia Giannopoulou^1^, Yuzhen Feng^3^, Wouter van den Braak^2^, Abbas Freydoonian^2^, Ramon Ramlal^2^, Hugo Meiring^2^, Belén Solano^1^, Wouter H. Roos^3^, Arjen J. Jakobi^1^, Leo A. van der Pol^2^, and Nynke H. Dekker^1,4,*^**

^1^ Delft University of Technology, Van der Maasweg 9, 2629 HZ Delft, The Netherlands

^2^ Intravacc B.V., Antonie van Leeuwenhoeklaan 9, 3721 MA Bilthoven, The Netherlands

^3^ Moleculaire Biofysica, Zernike Instituut, Rijksuniversiteit Groningen, Nijenborgh 4, 9747 AG Groningen, The Netherlands

^4^ *Department of Physics and Kavli Institute of Nanoscience Discovery, University of Oxford, Oxford, United Kingdom*

* *nynke.dekker@physics.ox.ac.uk*

This supplementary information consists of 6 supplementary figures and 3 supplementary tables describing:

- The EM workflow. **Related to Figure 2.**
- The structural comparison of our VLPs to published structures. **Related to Figure 2.**
- The MS analysis. **Related to Figure 2.**
- The mechanical parameters (spring constant, critical force, critical indentation, and Young modulus) measured by AFM. **Related to Figure 4**.
- The AFM comparative measurements for EV71 to the CVA6 VLPs and virions. **Related to Figure 4**.
- The AFM investigation into the influence of the inactivation process on the stability of the capsids. **Related to Figure 4**.
- The numerical values from the AFM measurements and used for Young moduli calculations. **Related to Figure 4**.

## Supplementary Figures

**Fig A. Cryo-EM processing workflow in Cryosparc.**

(A) Raw micrograph processing on CryoSPARC and steps required to proceed with 2D classification.

(B) 2D classes of selected particles.

(C) *Ab initio* reconstruction of cryo-EM density using Icosahedral symmetry.

(D) Homogeneous refinement of map that resulted in 3.15 Å resolution. The final density map obtained can be viewed in cyan through its 5-fold and 2-fold symmetry axes.

(E) Quality assessment metrics.  (upper left) GSFSC resolution as computed from the processing software CryoSPARC, indicates a final map resolution of 3.15 Å resolution.  (upper right) Local resolution of the final map plotted on the surface of the map as a colour gradient that ranges from 2.8-3.8 Å. (lower left) Euler angle distribution plot that shows the angular distribution of the particles (lower right) Model vs FSC plot evaluates the map-to-model fit match at 0.5 threshold. **Related to Figure 2.**

**Fig B. Evaluation of the increased density at the center of the VLPs.**

(A) Comparison of the cryo-EM density of the current study VLP (blue), EMD_6829 (yellow), EMD_6752 (green) and EMD_14186 (purple), shown from the face of the 5-fold axis, (1st column), the 2-fold axis (2^nd^ column), and the central slice of each particle (3^rd^ column).

(B) Graphical plot of the normalized radial intensity of each particle density plotted against the radius (Å).

(C) Comparison of the pdb models of the ASUs of the VLP originating from this study (blue), 5yhq (yellow), and 5xs5 (green) rotated along the x axis by 90° from left to right. **Related to Figure 2.**

**Fig C. LCMS analysis assessing the cleavage of the viral protein VP0 as indicator of viral maturation.**

(A) Full VP0 amino acid sequence, with VP4 and VP2 indicated in red and green, respectively. Bold and underlined indicates the concatenated VP4/VP2 sequence of the proteotypic peptide for VP0 upon digestion. For VLPs that underwent viral maturation, the C-terminal proteotypic peptide of VP4 (EVAAPLQ) and the N-terminal proteotypic peptide of VP2 (SPSVEACGYSDR) should be identified, while for immature VLPs, the concatenated sequence of these VP4-VP2 termini (EVAAPLQSPSVEACGYSDR) should be identified.

(B) MS analysis revealed the presence of VP0 (concatenated VP4-VP2) in high abundance. Additionally, the C-terminal and N-terminal proteotypic peptides of VP4 and VP2, respectively, were identified, however in extremely low concentrations (~3 orders of magnitude lower than that of VP0). **Related to Figure 2.**

**Fig D. AFM nanoindentation results for EV71, CVA6 virions and VLPs.**

(A) Corrected height of the particles (EV71 VLPs (black), EV71 virions (red), CVA6 VLPs (blue), and CVA6 virions (violet)) as determined by AFM measurements.

(B) Viral spring constant (N/m).

(C) Critical force (nN).

(D) Critical indentation (nm).

P values are indicated by asterisks: p<0.001 (***), p<0.01 (**), and p<0.05 (*). N.s.: not significant. The CVA6 data is presented again for direct comparison with the EV71 data. **Related to Figure 4.**

**Fig E. AFM particle stability parameters not affected by formaldehyde inactivation.**

To investigate whether stabilizing effects of formaldehyde treatment accounted for the observed differences in the mechanical properties between virions and VLPs, a supplementary batch of CVA6 VLPs underwent the same formaldehyde treatment as the CVA6 virions, after which we again performed nanoindentation force spectroscopy experiments using the AFM.

(A) Viral spring constant (N/m). CVA6 VLP (blue), CVA6 inactivated virions (violet), and CVA6 inactivated VLPs (green).

(B) Critical force (nN).

(C) Critical indentation (nm).

P values are indicated by asterisks: p<0.001 (***), p<0.01 (**), and p<0.05 (*). N.s.: not significant. From these experiments, we concluded from this data that there was no significant difference between treated and untreated CVA6 VLPs. The same is assumed to hold true for EV71 VLPs. Therefore, it was concluded that the inactivation process did not have any additional stabilizing effects on the CVA6 virion capsids. **Related to Figure 4.**

**Fig F. Mass photometry data on CVA6 VLPs.**

To further investigate the content of the CVA6 VLPs, mass photometry experiments were performed. The results revealed a widespread distribution, indicating heterogeneity in the VLP content. Four populations were identified (red Gaussian fits) which approximately correlated with the molecular weights of the empty particle (~3.8 MDa) and particles containing vRNA fragments coding for proteins 3C (~0.34 MDa; Gaussian peak at 4.2 MDa), 3D (~0.86 MDa; Gaussian peak at 4.6 MDa), and the P1 region (~1.59 MDa; Gaussian peak at 5.1 MDa). Differences observed for the P1 peak may have arisen from the Gaussian fits applied to the data and the limited statistical sample size.

## Supplementary Tables

**Table A. Measured size and determined mechanical parameters for the different capsid structures. Related to Figure 4.**

| Sample | Number of capsids | Size (mean ± SD; nm) | Spring constant (mean ± SD; N/m) | Critical force (mean ± SD; nN) | Critical indentation (mean ± SD; nm) |
| --- | --- | --- | --- | --- | --- |
| EV71 VLP | 47 | 30 ± 2 | 0.3 ± 0.1 | 0.6 ± 0.3 | 4 ± 2 |
| EV71 virion | 22 | 29 ± 2 | 0.3 ± 0.2 | 1.0 ± 0.5 | 6 ± 2 |
| CVA6 VLP | 48 | 29 ± 2 | 0.3 ± 0.2 | 0.7 ± 0.3 | 3 ± 1 |
| CVA6 virion | 11 | 30 ± 2 | 0.4 ± 0.2 | 1.12 ± 0.3 | 5 ± 1 |
| CVA6 inact. VLP | 35 | 28 ± 1 | 0.3 ± 0.2 | 0.7 ± 0.2 | 3 ± 1 |

**Table B. Deduction of Young’s moduli from measured size parameters for the different capsid structures. Related to Figure 4.**

| Sample | Shell thickness (nm) | Young modulus (mean ± SD; GPa) |
| --- | --- | --- |
| EV71 VLP | 4.5 | 0.2 ± 0.1 |
| EV71 virion | 5.0 | 0.17 ± 0.09 |
| CVA6 VLP | 4.9 | 0.2 ± 0.1 |
| CVA6 virion | 5.9 | 0.2 ± 0.1 |
| CVA6 inact. VLP | 4.9 | 0.2 ± 0.1 |

**Table C. Processing and structural information of our CVA6 VLP.**

| **CVA6 VLPs** | |
| --- | --- |
| **Data collection** | |
| Microscope | JEM 3200FSC |
| Voltage (kV) | 300 |
| Detector | K2 Summit |
| Energy Filter | In-column omega filter |
| Micrographs collected (No.) | 643 |
| Pixel size (Å) | 0.9751 |
| Electron exposure (e-/Å2) | 57.23 |
| Frame number | 45 |
| Exposure time (s) | 9 |
| Defocus range (μm) | -0.5 to -2.5 |
| **Data processing** | |
| Symmetry imposed | Icosahedral (I) |
| B-factor (Å2) | 132.6 |
| Final number of particles | 44,582 |
| Final map resolution (Å) | 3.15 |
| Map resolution range (Å) | 2.8-3.8 |
| **Model refinement** | |
| Initial model used (PDB code) | 5YHQ (derived from) |
| Primary sequence (Uniprot ID) | A0A0D3QLE1 |
| PDB ID | 8R6X |
| EMDB ID | EMD-18968 |
| **Model resolution** | |
| FSC map-model (0.5) | 3.45 |
| Map sharpening B-factor (Å2) | -132.6 |
| Model composition  Non-hydrogen atoms   Protein residues | 4875  622 |
| **B-factors (Å2)**  Overall (Å2)  Protein (Å2) | 130.3  130.3 |
| **Validation** MolProbity score Clashscore | 1.82  5.72 |
| RMSD  Bond lengths (Å)  Bond Angles (°) | 0.0045  1.02 |
| Rotamer outliers (%) | 1.92 |
| Ramachandran plot  Favoured (%)  Allowed (%)  Disallowed (%) | 94.41  5.59  0 |

Fig A

Fig B


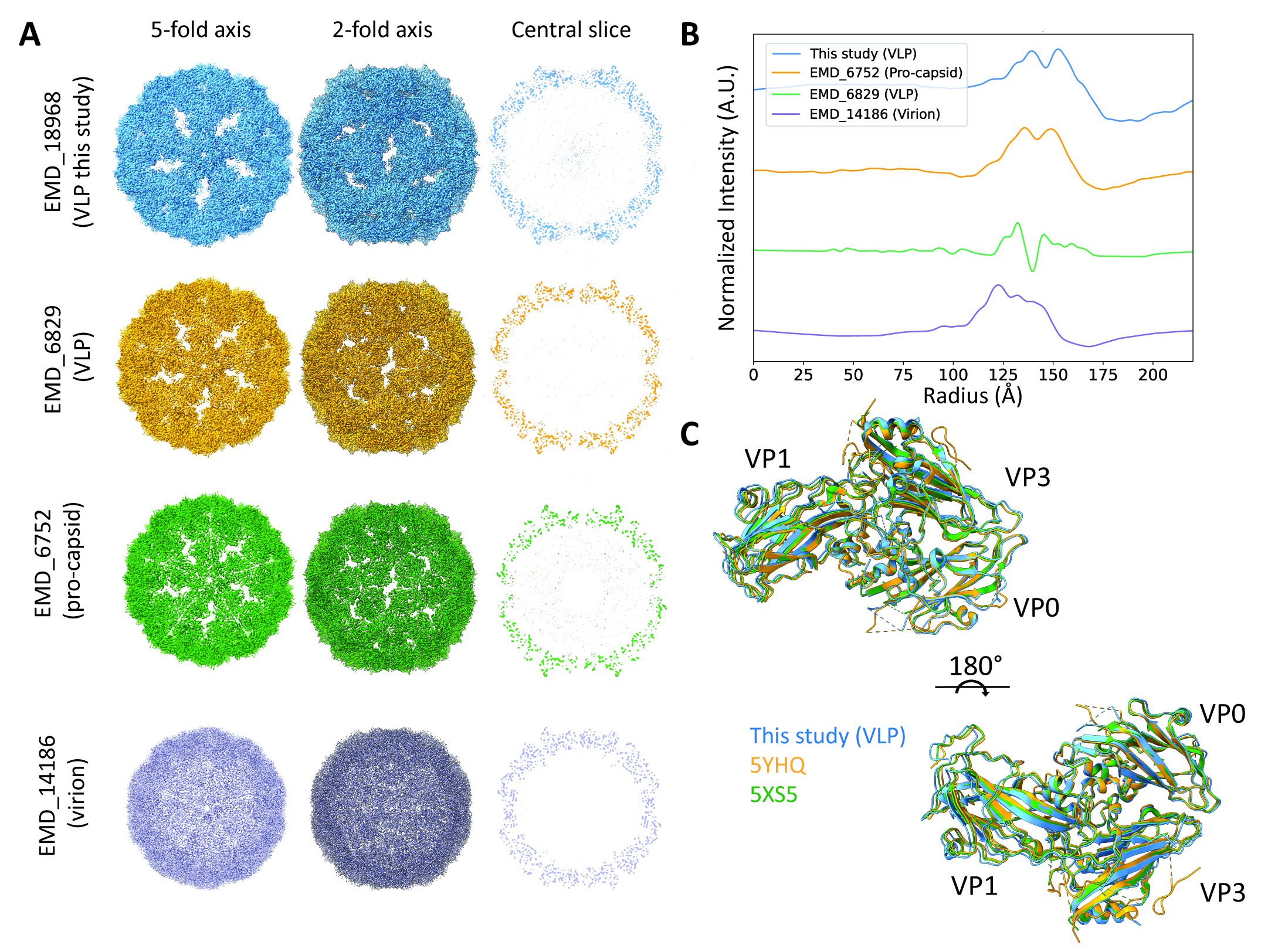


Fig C


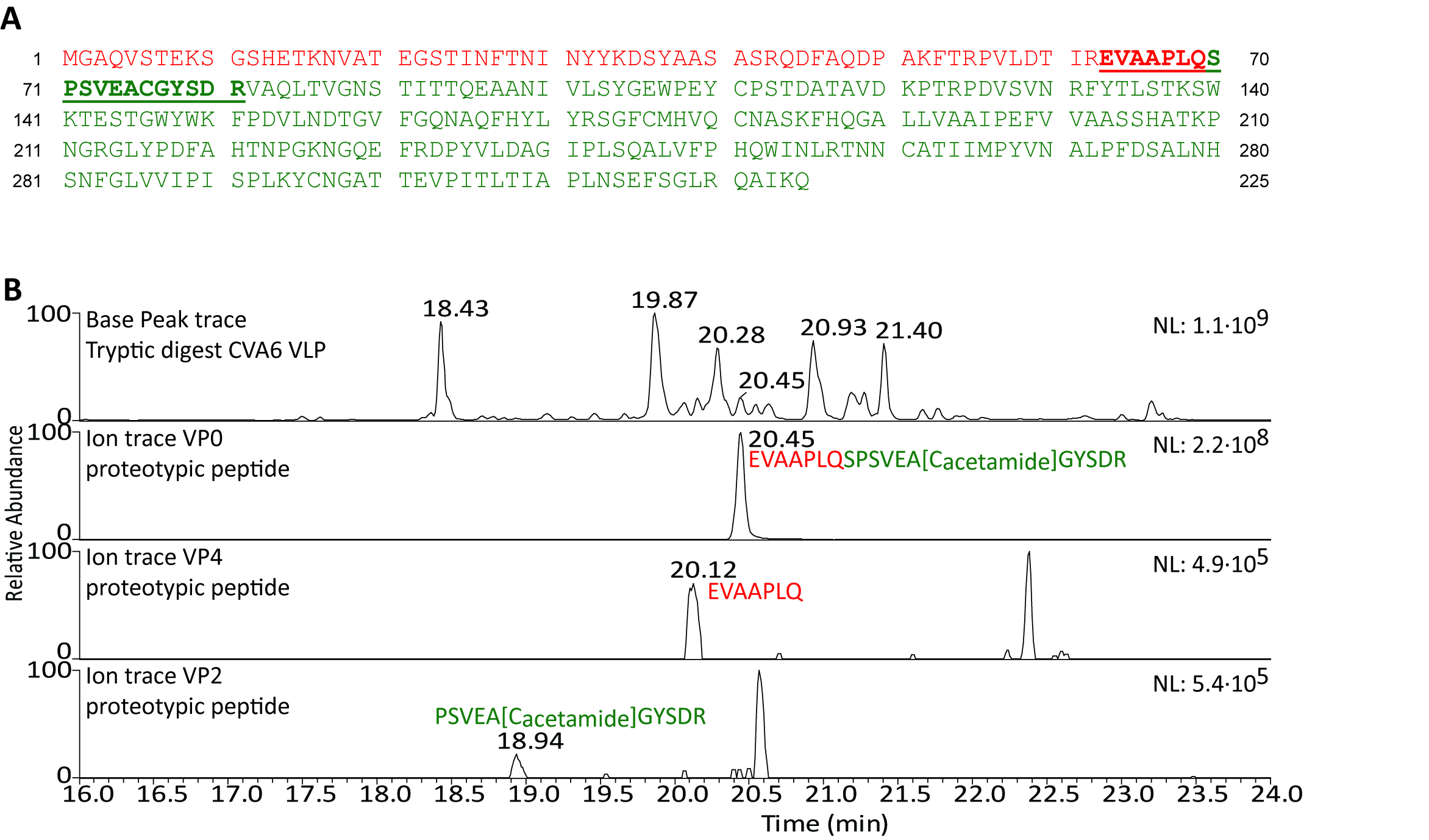


Fig D


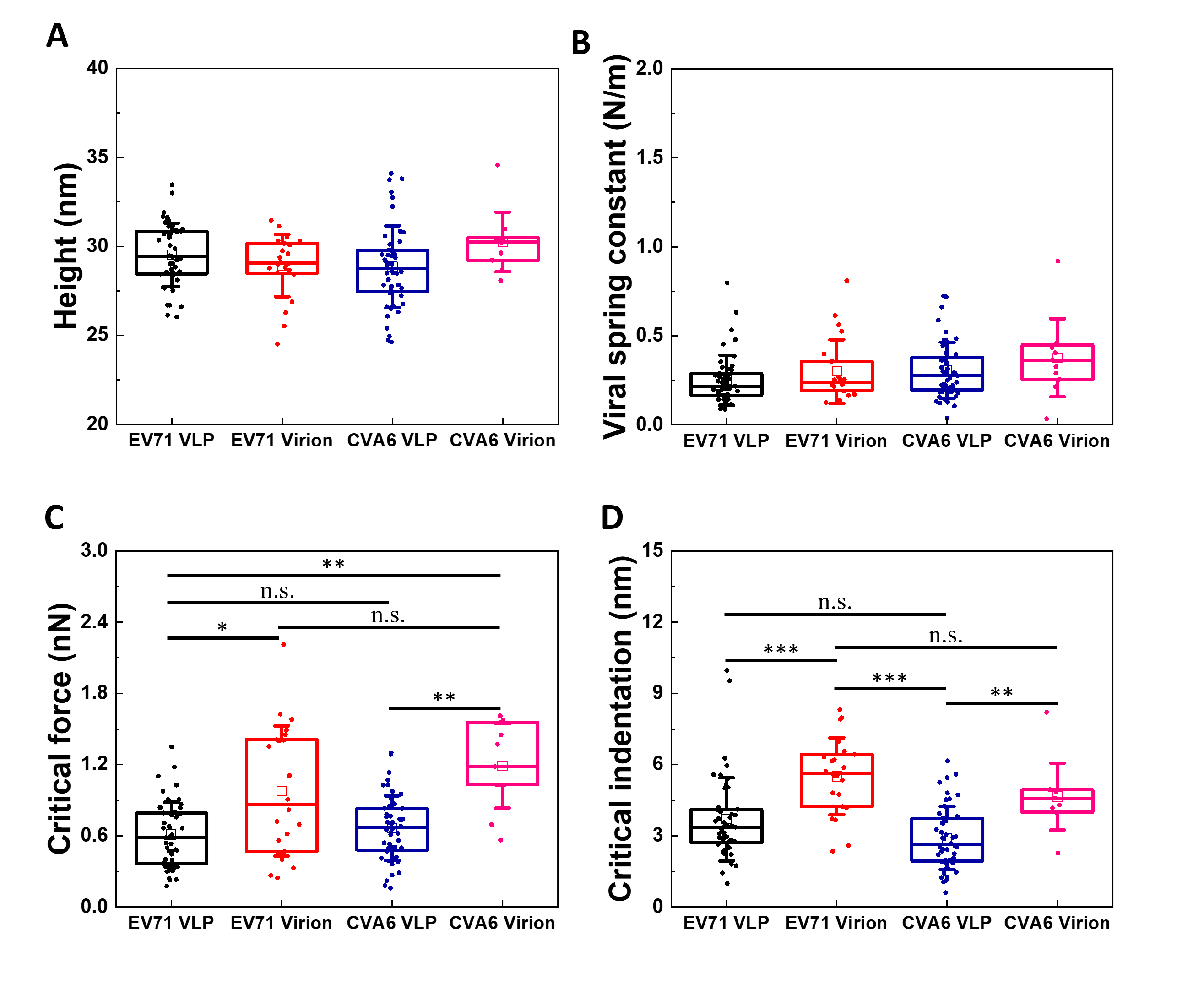


Fig E


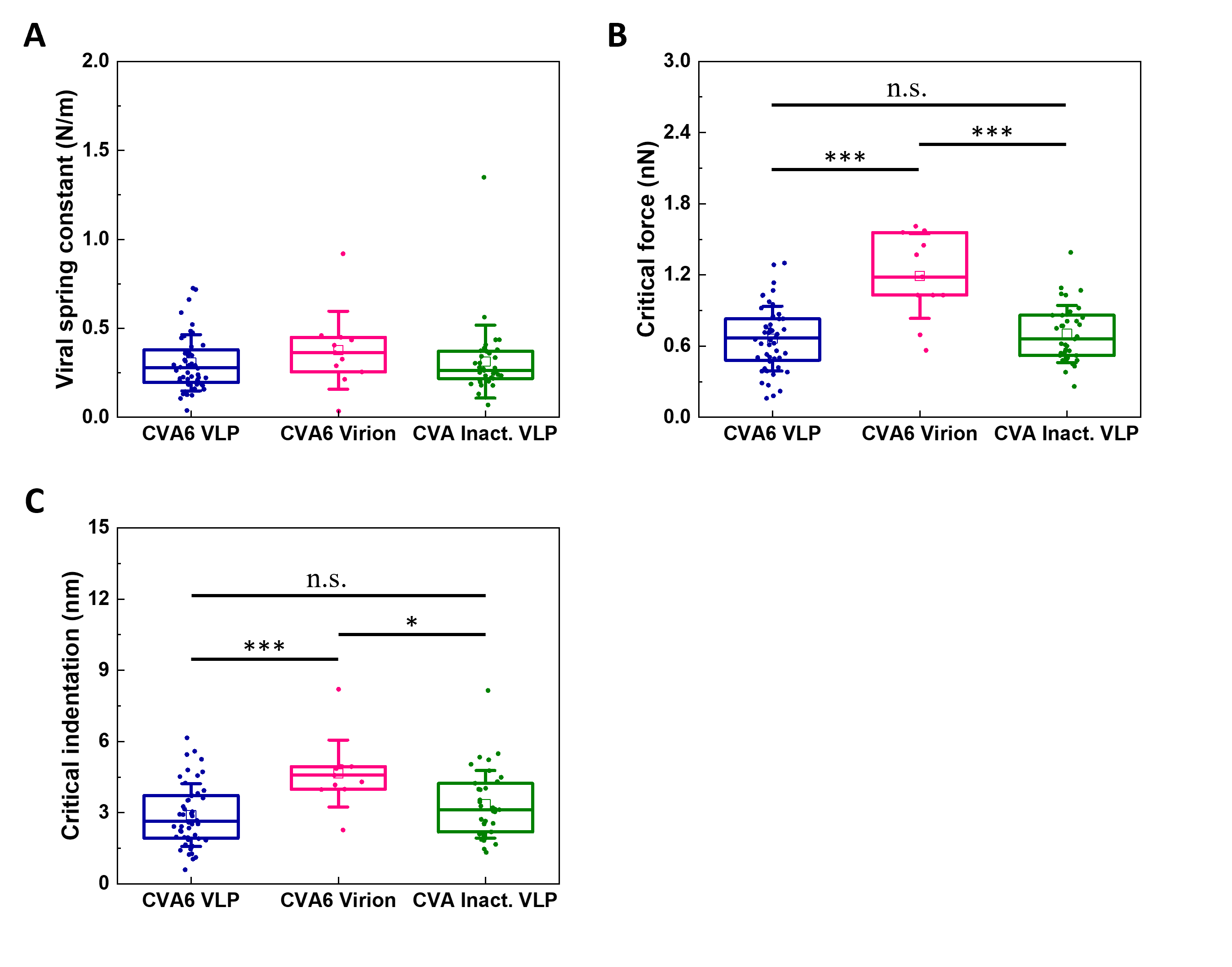


Fig F


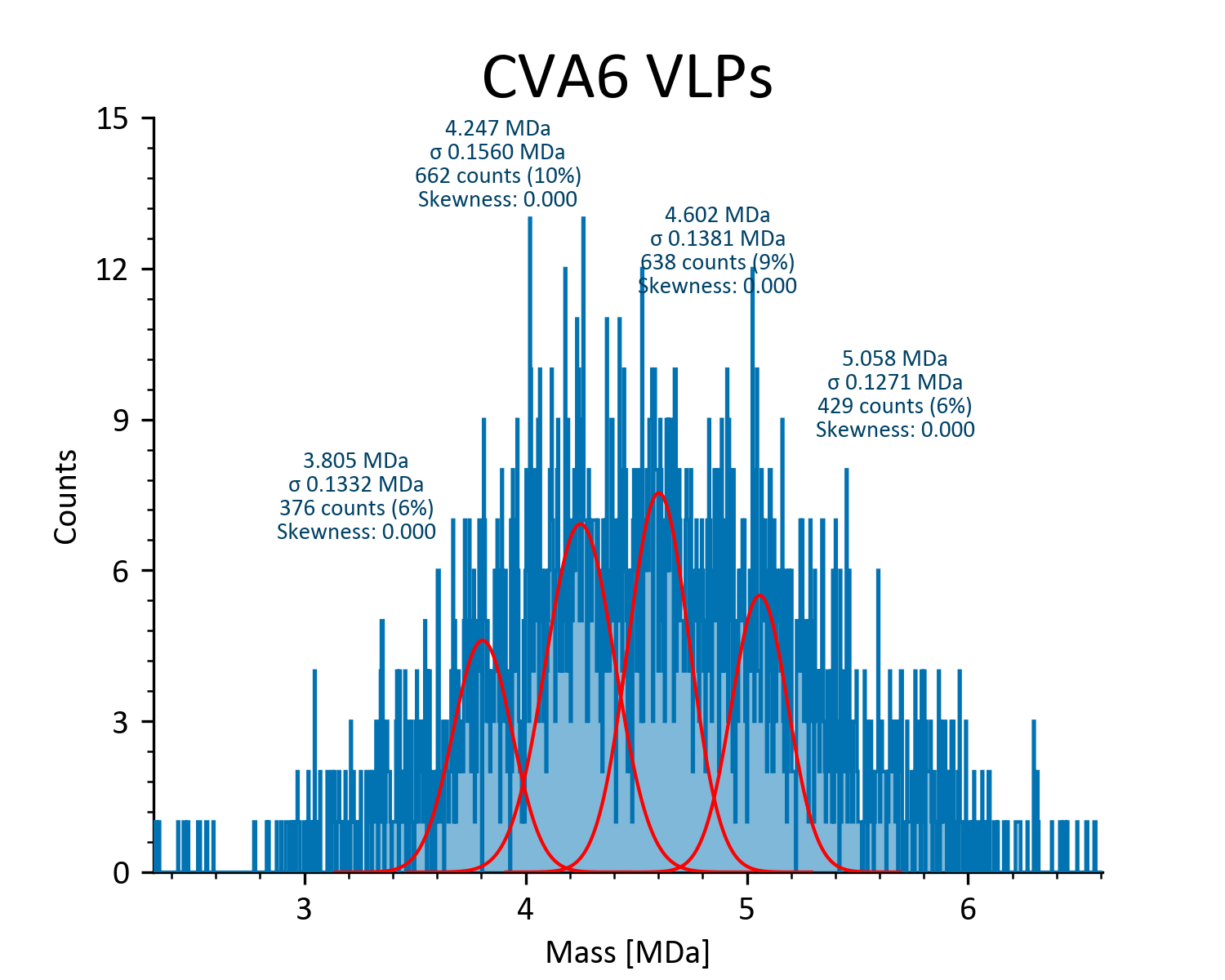

Supplement: S1 Text — Fig A. Cryo-EM processing workflow in Cryosparc. (A) Raw micrograph processing on CryoSPARC and steps required to proceed with 2D classification. (B) 2D classes of selected particles. (C) Ab initio reconstruction of cryo-EM density using Icosahedral symmetry. (D) Homogeneous refinement of map that resulted in 3.15 Å resolution. The final density map obtained can be viewed in cyan through its 5-fold and 2-fold symmetry axes. (E) Quality assessment metrics. (upper left) GSFSC resolution as computed from the processing software CryoSPARC, indicates a final map resolution of 3.15 Å resolution. (upper right) Local resolution of the final map plotted on the surface of the map as a colour gradient that ranges from 2.8–3.8 Å. (lower left) Euler angle distribution plot that shows the angular distribution of the particles (lower right) Model vs FSC plot evaluates the map-to-model fit match at 0.5 threshold. Related to Fig 2. Fig B. Evaluation of the increased density at the center of the VLPs. (A) Comparison of the cryo-EM density of the current study VLP (blue), EMD_6829 (yellow), EMD_6752 (green) and EMD_14186 (purple), shown from the face of the 5-fold axis, (1st column), the 2-fold axis (2nd column), and the central slice of each particle (3rd column). (B) Graphical plot of the normalized radial intensity of each particle density plotted against the radius (Å). (C) Comparison of the pdb models of the ASUs of the VLP originating from this study (blue), 5yhq (yellow), and 5xs5 (green) rotated along the x axis by 90° from left to right. Related to Fig 2. Fig C. LCMS analysis assessing the cleavage of the viral protein VP0 as indicator of viral maturation. (A) Full VP0 amino acid sequence, with VP4 and VP2 indicated in red and green, respectively. Bold and underlined indicates the concatenated VP4/VP2 sequence of the proteotypic peptide for VP0 upon digestion. For VLPs that underwent viral maturation, the C-terminal proteotypic peptide of VP4 (EVAAPLQ) and the N-terminal prot [file ppat.1012873.s001.docx]
